# Knowledge Assessment Regarding the Differences Between Hypothyroidism and Hyperthyroidism Among Saudi Arabia’s Adult Population

**DOI:** 10.7759/cureus.37830

**Published:** 2023-04-19

**Authors:** Hyder Mirghani, Sarah M Alquayr, Tahani Alanazi, Abdulaziz A Alwakeel, Abdullah M Al Madshush, Hatoun A Alali, Ghassan S Alerrwi, Abdullah D Alshehri

**Affiliations:** 1 Internal Medicine, University of Tabuk, Tabuk, SAU; 2 Medicine, University of Tabuk, Tabuk, SAU

**Keywords:** hypothyroidism, hyperthyroidism, thyroid disease, saudi arabia, assessment, knowledge, differences

## Abstract

Introduction

Worldwide, thyroid diseases are among the most prevalent endocrine disorders. According to the Saudi Arabian Ministry of Health (MOH), many thyroid disease cases remain undiagnosed and, as a result, are not treated because the patient has no symptoms or is unaware of them. Hence, this study aims to assess the knowledge about hypothyroidism and hyperthyroidism among Saudi Arabia's population.

Methodology

A cross-sectional survey was conducted among Saudi adults in five randomly selected regions in Saudi Arabia from December 2022 - January 2023. An Arabic self-administered questionnaire was sent to randomly selected participants via an online link. The questionnaire was composed of four parts: Sociodemographic; knowledge related to hypothyroidism and hyperthyroidism diseases and their differences; knowledge about the thyroid gland in terms of functions and causes of thyroid dysfunction. The Statistical Package for Social Sciences was used for data analysis.

Results

Out of 996 participants (66.2% women), 70.1% knew the function of the thyroid gland, 66.4% knew that women are more susceptible to thyroid disease, and 49.5% knew the association between thyroid dysfunction and heart disease. Female sex, higher education, and old age were associated with good knowledge, and no differences were evident regarding nationality and residence. The results showed inadequate awareness regarding thyroid diseases in Saudi Arabia, with some parts of this population being very clearly below average.

Conclusion

Knowledge regarding thyroid disorders was sub-optimal in Saudi Arabia; older women with higher education had the best knowledge. With even larger samples, we recommend that future studies be made to develop clear and decisive public health strategies that can be implemented at once.

## Introduction

Worldwide, thyroid diseases are among the most prevalent endocrine disorders [1]. The thyroid gland, situated in the front of the neck, is thought to be the largest endocrine gland in the human body. It produces and secretes thyroid hormones, significantly impacting protein synthesis and basal metabolic rate (BMR) [1]. The growth of tissues and organs is another function of thyroid hormones. Thyroid-stimulating hormone (TSH) from the anterior pituitary gland and thyroid-releasing hormone from the brain control thyroid hormone production [2]. Hyperthyroidism is defined by increased thyroid hormone production and thyroid gland secretion. The leading cause of hyperthyroidism is Graves' disease [3]. Thyroid hormone synthesis is reduced in hypothyroidism, which can be primary (caused by a defect in the thyroid gland) or secondary (caused by pituitary or hypothalamic illness). Nearly all cases of hypothyroidism (about 99%) are caused by primary hypothyroidism [4].

According to the Saudi Arabian Ministry of Health (MOH), many thyroid disease cases remain undiagnosed and, as a result, are not treated because the patient has no symptoms or is unaware of them [4]. Patients with thyroid diseases may exhibit a wide range of symptoms during their clinical evaluation, including those involving the endocrine, cardiovascular, central nervous, musculoskeletal, hematological, reproductive, gastrointestinal, and dermatological systems of the body [5]. Thyroid function test panels are frequently used for diagnosing and assessing thyroid diseases. According to the American Thyroid Association, adults must start thyroid screening using serum TSH at age 35 and quinquennially after that [6].

Both hyperthyroidism and hypothyroidism affect 2% and 1% of people, respectively. The incidence in men is roughly one-tenth that in women [7]. Studies have been published on the knowledge and perception of hyper-hypothyroidism. In 2010, a study was conducted among patients with hypothyroidism in community hospital endocrine clinics in Chennai. The study found that many hypothyroid patients had inadequate knowledge about their condition, including misconceptions about terminologies, symptoms, and treatment. Some patients had false information, such as iodized salt could cure hypothyroidism. Dietary restrictions and medication use also posed challenges, with some patients adhering to unnecessary restrictions and misconceptions about when to stop the medication. Only a small percentage knew about alternative treatments for hypothyroidism [8].

In 2016, a knowledge and awareness study regarding thyroid disorders was assessed in India. In the study, 29.2% of females had never heard the word "thyroid," 48.8% of females had excessive sensitivity to heat or cold, 25.2% had long-term constipation or diarrhea, 61.2% had joint or muscle pain/weakness, 69.6% had anxiety/depression/mood swings, 39.6% had irregular menstruation, 82.4% of females had a problem with hair loss/skin changes. Only 49.20% were aware of hyperthyroidism and hypothyroidism, 25.2% had a thyroid screening test, and 55.2% believed alternative therapies might treat thyroid diseases [9].

In 2018, a previous study assessed the knowledge regarding the difference between hypothyroidism and hyperthyroidism of 300 adult residents in Tabuk City, Saudi Arabia. An Arabic self-administered questionnaire was used. The questionnaire contained questions about sociodemographic data and other questions to assess knowledge about the thyroid gland. The result showed that 52% had good knowledge of the thyroid gland and its disorders, while 45% had poor knowledge. Answers to questions about the type of thyroid gland 71.4% and the people most likely to have the disorder showed good knowledge 90.4%. Most respondents also acknowledged hypothyroidism symptoms, including weight gain 76%, irritability, and tiredness 74.9%. Regarding the functioning of the thyroid gland, the causes of thyroid hormone abnormalities, and the signs of hyperthyroidism, inadequate and poor knowledge was discovered [10].

In 2021, another study was conducted regarding the knowledge of thyroid disease manifestation and risk factors. A cross-sectional study was conducted with 882 randomly selected participants in the Eastern Saudi Arabian province from July 2020 to October 2020. Overall, 44.7%, 41.2%, and 14.2% were categorized as having low, average, and high knowledge, respectively. The mean knowledge score was 8.67. The study demonstrates that having a better knowledge of thyroid diseases was significantly more associated with being female, living in Al Ahsa, being a student, having a prior history of thyroid disease, having a family history of the disease, and having their thyroid gland examined. Regarding thyroid disease manifestations and associated risk factors, only half of the group under study scored highly on awareness [1].

Conducting such studies on public knowledge regarding the difference between hypothyroidism and hyperthyroidism can play a significant role in helping physicians concentrate on specific issues during the first interaction with patients and strategize future activities to improve disease outcomes. Hence, this study aims to assess the knowledge level about hypothyroidism and hyperthyroidism among Saudi Arabia's population.

## Materials and methods

Study design

After approval by King Fahad Medical Center Ethical Committee Approved No. (22-600E), The research design used to conduct this study was an observational cross-sectional study based on a structured online questionnaire developed by Alhawiti et al., 2018 [10]. It was carried out from December 2022 - January 2023 and was conducted on adults.

Settings and target population

The study area was Saudi Arabia. It was conducted in the five regions of Saudi Arabia (Eastern, Central, Northern, Western, and Southern regions), with a population of 34.81 million (2020). Adults living in the Kingdom of Saudi Arabia were the targeted population.

Sampling techniques and sample size

Simple random sampling techniques were used in the study. The participants were randomly selected via social media and requested to complete an Arabic self-administered online questionnaire. The Qualtrics calculator was used to estimate the sample size of 385 with a 95% confidence level. To produce a representative survey sample with less bias, an additional 150.91% was included. 996 of the 1040 individuals from Saudi Arabia participated after some were excluded (based on exclusion criteria).

Inclusion criteria: Adults living in Saudi Arabia within the five regions, of all sexes and nationalities, and who are at least 18 years old, with their consent to participate. Participants under 18, people working in the health industry, and those who declined to participate were excluded.

Preparing the study instrument

Following a thorough literature analysis, An Arabic self-administered questionnaire was sent to randomly selected participants via an online link. The questionnaire was composed of four parts: (1) Sociodemographic questions, (2) Questions related to knowledge of hypothyroidism and hyperthyroidism diseases and the differences between them, (3) Knowledge about the thyroid gland in terms of functions and causes of thyroid dysfunction, and (4) Respondents who have or suspect thyroid disease. The data was collected from December 2022 - January 2023.

Statistical analyses

After data was collected via Google Forms, "Microsoft Excel Software™" entered the data on the computer. Then it transferred to the IBM Statistical Package of Social Science Software (IBM SPSS™) program, version 20, for statistical analysis.

## Results

A total of 996 individuals responded to the study questionnaire. Of the survey participants, nearly twice as many females responded compared to males, and nearly all participants were born in Saudi Arabia. Most participants were from the Western region of Saudi Arabia, while the fewest lived in the Central region. Approximately half of the participants were 18 to 35 years old, and the other half were greater than 35 years old. Half the participants were married, and approximately three-quarters lived in the city.

Furthermore, nearly two-thirds of respondents had a Bachelor's degree, nearly 25% had a high school diploma only, and nearly 10% had a graduate degree. Ranked from highest to lowest, the remaining 5% of participants had a secondary degree, elementary-level education, or no degree. Approximately one-third of the participants were students, and slightly over one-third were employed. In descending order, 30% of participants were homemakers, unemployed, or retired (Table 1). 

**Table 1:** Socio-demographic data of the survey respondents.

(N = 996)	n (%)
Gender	
Female	659 (66.2)
Male	337 (33.8)
Age	
18-35	526 (52.8)
36-50	347 (34.8)
>50	123 (12.4)
Nationality	
Saudi	954 (95.8)
Non-Saudi	42 (4.2)
Region	
Central region	99 (9.9)
Northern region	220 (22.1)
Eastern region	237 (23.8)
Southern region	165 (16.6)
Western region	275 (27.6)
Lives in	
rural	266 (26.7)
City	730 (73.3)
Marital status	
unmarried	424 (42.6)
married	504 (50.6)
Separated/Divorced/Widowed	68 (6.8)
Educational Level	
none	9 (0.9)
Elementary school	12 (1.2)
Secondary school	30 (3.0)
High school diploma	221 (22.2)
Bachelor’s degree	627 (63.0)
Graduate degree	97 (9.7)
Occupation	
unemployed	101 (10.1)
employed	386 (38.8)
housewife	128 (12.9)
student	317 (31.8)
retired	64 (6.4)

Nearly three-quarters of participants understood what a thyroid gland is and its function. Moreover, two-thirds of participants understood that women are likelier to have thyroid dysfunctions than men. Despite these understandings, appropriately half of the respondents needed to understand that thyroid dysfunction can lead to heart disease and influence brain development and cholesterol levels. Moreover, under half of the participants understood that thyroid dysfunctions are hereditary (Table 2).

**Table 2:** Knowledge about the thyroid gland and causes of thyroid disease.

(N = 996) n (%)			n (%)
Thyroid is a ductless (endocrine) gland?		Foods that have a good effect on thyroid function include?	
no idea	236 (23.7)	Iodine-rich food	614 (61.6)
no	63 (6.3)	Protein-rich food	211 (21.2)
yes	697 (70.0)	None of the above	171 (17.2)
Who is more susceptible to have thyroid dysfunctions?		Thyroid dysfunction affects brain development?	
children	77 (7.7)	no idea	387 (38.9)
elderly	196 (19.7)	no	149 (15.0)
men	62 (6.2)	yes	460 (46.1)
women	661 (66.4)		
Functions of thyroid include?		Thyroid dysfunction affects blood cholesterol level?	
Enhancing metabolism	241 (24.2)	no idea	359 (36.0)
Growth and development of fetal neurological system	17 (1.7)	no	103 (10.4)
Regulation of heartbeats	40 (4.0)	yes	534 (53.6)
All of the above	698 (70.1)		
Thyroid dysfunction results in heart diseases?		Is there a confirmed relationship between smoking and thyroid disturbances?	
no idea	376 (37.8)	no idea	450 (45.2)
no	126 (12.7)	no	165 (16.6)
yes	494 (49.5)	yes	381 (38.2)
Thyroid dysfunctions are hereditary?			
no idea	268 (26.9)		
no	279 (28.0)		
yes	449 (45.1)		

Based on the percentage of correct responses, over half of the participants knew about most thyroid dysfunction symptoms. However, slightly under half of respondents identified oligomenorrhea and amenorrhea as a symptom of hyperthyroidism. Considering hypothyroidism, sudden weight gain was the most identified symptom. Compared to sudden weight gain, 20% fewer participants identified hair dryness as a symptom of hypothyroidism. Nearly 20% of participants had thyroid dysfunction. Over three-quarters of these participants had hypothyroidism, followed by slightly under one-fifth of participants having hyperthyroidism. Less than 1% of participants had thyroid cancer. Most respondents with thyroid dysfunction had consulted doctors for treatment and received medications for treatment. Of the participants not diagnosed with thyroid disease, slightly more than 10% suspected they had thyroid dysfunction, and approximately one-third of these individuals listed laziness as the most likely cause (Table 3).

**Table 3:** Respondent knowledge of hyper- and hypothyroidism symptoms.

(N = 996) n (%)			n (%)
Loss of weight despite good appetite is a symptom of hyperthyroidism?		Insomnia and lack of sleep are symptoms of hyperthyroidism?	
no idea	110 (11.0)	no idea	174 (17.5)
no	117 (11.7)	no	75 (7.5)
yes	769 (77.3)	yes	747 (75.0)
Increased heart rate is a symptom of hyperthyroidism?		Oligomenorrhea and amenorrhea are symptoms of hyperthyroidism?	
no idea	290 (29.1)	no idea	375 (37.7)
no	112 (11.2)	no	138 (13.9)
yes	594 (59.7)	yes	483 (48.4)
A sudden increase of weight is a symptom of hypothyroidism?		Fatigability and sleepiness are manifestations of hypothyroidism?	
no idea	147 (14.8)	no idea	194 (19.5)
no	55 (5.5)	no	70 (7.0)
yes	794 (79.7)	yes	732 (73.5)
Skin and hair dryness are symptoms of hypothyroidism?		Feeling cold in hot weather is a symptom of hypothyroidism?	
no idea	306 (30.7)	no idea	267 (26.8)
no	84 (8.5)	no	114 (11.4)
yes	606 (60.8)	yes	615 (61.8)
Do you have thyroid disease?			
no	815 (81.8)		
yes	181 (18.2)		
If yes: (n=181)		If no: (n=815)	
what is the complaint?		do you suspect that you have thyroid disease?	
Hyperthyroidism	29 (16.0)	no	718 (88.1)
Hypothyroidism	151 (83.4)	yes	97 (11.9)
Thyroid cancer	1 (0.6)	If yes, why do you suspect?	
At which age this problem started?		A relative had the disease	14 (14.6)
1 – 12 years	7 (3.9)	Increased weight	20 (20.8)
12 – 20 years	21 (11.6)	Laziness	36 (37.5)
20 – 35 years	82 (45.3)	Weight loss	9 (9.4)
>35 years	71 (39.2)	One of these symptoms appeared	17 (17.7)
Do you consult a doctor for treatment?			
no	24 (13.3)		
yes	157 (86.7)		
What are the treatments you take?			
dietary	50 (27.6)		
medications	131 (72.4)		

The median knowledge score regarding a participant's understanding of the differences between hypothyroidism and hyperthyroidism was 25.7. Participants with scores above this median were considered to have good levels of knowledge, while those below the median had poor levels of knowledge. Slightly over half of the participants had good levels of knowledge, and slightly under half had poor knowledge. Approximately 20% more female participants had good knowledge levels than males (p= <0.001). Knowledge level was also significantly associated with age group (p=0.040), educational level (p=0.000), and region (p=0.002). Specifically, compared to the 18-35 and >50-year-old age groups, more individuals within the 36-50 age group were classified as having poor knowledge regarding the differences between hypothyroidism and hyperthyroidism. Individuals with a high school diploma or higher-level degrees had greater knowledge than those without degrees, and knowledge levels were greatest for individuals with a bachelor's or graduate degree. Participants within the central and eastern regions had greater knowledge than other regions. No associations were found between knowledge level and marital status, occupation, nationality, or where the participants lived (Table 4).

**Table 4:** Associations between participant characteristics and their knowledge about differences between hypothyroidism and hyperthyroidism.

Variables	Knowledge		P-value
	poor knowledge (n=453, 45.5%)	good knowledge (n=543, 54.5%)	
Age (Years)			0.040†‡
18-35	247 (47.0)	279 (53.0)	
36-50	141 (40.6)	206 (59.4)	
>50	65 (52.8)	58 (47.2)	
Gender			0.000†‡
Female	256 (38.8)	403 (61.2)	
Male	197 (58.46)	140 (41.54)	
Marital status			0.699†
Unmarried	194 (45.7)	230 (54.3)	
Married	225 (44.6)	279 (55.4)	
Separated/Divorced/Widowed	34 (50.0)	34 (50.0)	
Occupation			0.050†
Unemployed	55 (54.4)	46 (45.6)	
Employed	183 (47.4)	203 (52.6)	
Housewife	62 (48.4)	66 (51.6)	
Student	124 (39.1)	193 (60.9)	
Retired	29 (45.3)	35 (54.7)	
Educational Level			0.000†‡
none	8 (88.9)	1 (11.1)	
Elementary school	10 (83.3)	2 (16.7)	
Secondary school	22 (73.3)	8 (26.7)	
High school diploma	118 (53.4)	103 (46.6)	
Bachelor’s degree	253 (40.3)	374 (59.7)	
Graduate degree	42 (43.3)	55 (56.7)	
Nationality			0.121†
Non-Saudi	24 (57.1)	18 (42.9)	
Saudi	429 (45.0)	525 (55.0)	
Lives in			0.474†
Rural	116 (43.6)	150 (56.4)	
City	337 (46.2)	393 (53.8)	
Region			0.002†‡
Central region	55 (55.6)	44 (44.4)	
Northern region	90 (40.9)	130 (59.1)	
Eastern region	128 (54.0)	109 (46.0)	
Southern region	64 (38.8)	101 (61.2)	
Western region	116 (42.9)	159 (57.1)	

## Discussion

Thyroid disorders are on the rise globally [11], and certain strata of society are more heavily affected than others [12]. The Kingdom of Saudi Arabia is not an exception; therefore, we aimed to conduct this study to have an up-to-date overview of the thyroid disease burden in this country and to have the necessary data to tackle the points of weakness that allow for an accelerated deterioration of the situation.

It comes immediately notice that awareness is correlated with specific sociodemographic characteristics. In our study, 61.2% of the female participants had good knowledge of the subject, while only 41.5% of the male participants had good knowledge. This can be attributed to the higher incidence among women [7]. The age group with the highest level of knowledge was between 36 and 50 years old, while the lowest was the group older than 50. The marital status does not seem to influence the awareness level significantly; however, education level is strikingly correlated, as 60% of holders of Bachelor's degrees had good knowledge, as well as 57% of Graduate degree holders, as opposed to only 11% of those with no education, and 17% of those at elementary school level. Occupation plays a role, too, as students had the best level of awareness at 61%, while the unemployed had the worst at 46%. Geographically speaking, our data showed no significant difference between those who live in rural or urban areas. However, inhabitants of KSA's central and eastern regions had lower levels of knowledge than other regions, at 44% and 46%, respectively. This comes reasonably expected, as a previous study conducted in KSA showed similar correlations; females had better knowledge than males (56% compared to 44%), the unemployed had the worst level of knowledge at 7.6%, and the marital status did not have a significant correlation [3].

Delving deeper into the gland and its disorders, it should be commonly known that the thyroid gland is an endocrine gland, sometimes described as "ductless". The physiologic function of this gland affects every organ system and might present in a wide array of symptoms ranging from cardiologic and neurologic to musculoskeletal and psychiatric [13].

Because of the countless possible presentations of thyroid diseases, an adequate level of awareness plays a key role in early diagnosis, which might ensure a higher quality of life, fewer complications and comorbidities, and, overall, lower disease burden.

In the present study, 70% of the participants knew that the thyroid is an endocrine gland, and 66% knew that women are more susceptible to its diseases. Also, 70% knew that the gland's functions affect metabolism, fetal growth, and the neurologic system. However, only 49.5% knew these diseases might manifest with cardiac symptoms. Also, it is quite concerning that only 45% of the participants knew that thyroid disorders might be hereditary, 46% knew that they could affect brain development, and 38% knew that smoking could be related to thyroid disorders. Another study by Alhawiti et al. found that about 52% of respondents had good knowledge about the thyroid gland and its disorders, while 45% had poor knowledge. A high level of knowledge was shown when asked about the type of thyroid gland 71.4% and the people most likely to develop the disorder 90.4%. Regarding the functions of the thyroid gland, the causes of thyroid hormonal disturbances, and the signs of hyperthyroidism, inadequate and poor knowledge was discovered [10].

When asked about specific manifestations, 60% of participants knew that tachycardia might be a symptom of hyperthyroidism, 48% knew that menstrual irregularities might also be a symptom, and 61% knew that feeling cold might be a symptom too. A few other symptoms were more widely known, such as weight increase in hypothyroidism which was known by 80%, and insomnia in hyperthyroidism which was known by 75%. All of these concerning statistics of low awareness levels are also expected and confirm the previous studies, including a study published in 2018 which found very low levels of awareness regarding symptoms of thyroid diseases, where, for example, only 17% of participants knew that thyroid disorders might affect the menstrual cycle and 27% linked them with fatigue and weight loss [2].

It should be noted that 18.2% of the participants reported having thyroid disease, 83% reportedly had hypothyroidism, and 16% had hyperthyroidism. The age group with the highest incidence rate is 20-35, and 87% consult a doctor for treatment.

Our study has a relatively large sample of 996 participants. We tried to ensure that we covered all possible sociodemographic backgrounds; therefore, these results can be beneficial in guiding future public health policies. However, the responses are self-reported, which makes them prone to bias.

The problem with hyperthyroidism and hypothyroidism is that they are associated with many comorbidities, such as hypertension and diabetes mellitus, which makes them notorious for affecting the patient's quality of life. Additionally, the problem is not limited to the individual level. These comorbidities pose an unneglectable source of additional healthcare expenditures, shifting the available resources away from the domains that most need them [14,15].

Our study has limitations. This study depended on recall for questionnaire filling, so it was subject to recall bias. In addition, questionnaires distributed online restricted respondents to those with Internet access.

## Conclusions

Our study's results indicate an inadequate level of awareness regarding thyroid diseases in the population of Saudi Arabia, and some individuals demonstrated awareness levels that were below average. These findings are consistent with previous studies and underscore the urgent need to develop public health policies to address this issue.

In particular, we recommend launching targeted awareness campaigns focusing on groups at the highest risk and with the lowest levels of awareness. Such campaigns can help to limit the detrimental effects of thyroid diseases and promote early detection and treatment. While there is much work to be done, this is of utmost importance for the health and well-being of the population.

To this end, we recommend that future studies be conducted with even larger samples to develop clear and decisive public health strategies that can be implemented immediately. By leveraging the results of our study and others like it, we can take meaningful steps toward improving awareness and reducing the burden of thyroid diseases in Saudi Arabia.

## Appendices

(I) Socio-demographic data of the respondents:

1.     Age (Years):

●      <18

●      18-35

●      36-50

●      >50

2.     Are you currently:

●      Married

●      Separated/Divorced/Widowed

3.     Sex

●      Male

●      Female

4.     Educational Level

●      Graduate degree

●      Bachelor degree

●      High school diploma

●      Secondary school

●      Elementary school

●      None

5.     Occupation

●      Employed

●      Unemployed

●      Student

●      Working in medical field

●      Housewife

●      Retired

6.     Nationality

●      Saudi

●      Non-Saudi

7.     Lives in

●      Rural

●      City

8.     Region

●      Eastern region

●      Central region

●      Western region

●      Northern region

●      Southern region

(II) Knowledge about thyroid gland, its functions, and causes of thyroid disease

9.     Thyroid is a ductless (endocrine) gland:

●       Yes

●      No

10.     Who is more susceptible to have thyroid dysfunctions?

●      Children     

●      Men      

●      Women     

●      Elderly

11.  Functions of thyroid include…

●      Enhancing metabolism

●      Regulation of heartbeats

●      Growth and development of fetal neurological system

●      All of the above

12.  Foods that have a good effect on thyroid function include…

●      Protein-rich food

●      Protein-rich food

●      Iodine-rich food

●      None of the above

13.  Thyroid dysfunction affects brain development

●      Yes

●      No

14.  Thyroid dysfunction affects blood cholesterol level

●      Yes

●      No

15.  Thyroid dysfunction results in heart diseases

●      Yes

●      No

16.  Does sport affect thyroid dysfunction?

●      Yes

●      No

17.  Is there a confirmed relationship between smoking and thyroid disturbances?

●      Yes

●      No

18.  Thyroid dysfunctions are hereditary

●      Yes

●      No

(III) Knowledge of respondents about symptoms of hyper- and hypothyroidism

19.  Loss of weight despite good appetite is a symptom of hyperthyroidism

●      Yes

●      No

20.  Insomnia and lack of sleep are symptoms of hyperthyroidism

●      Yes

●      No

21.  Increased heart rate is a symptom of hyperthyroidism

●      Yes

●      No

22.  Inability to stand hot weather and wearing light clothes in cold weather are symptoms of hyperthyroidism

●      Yes

●      No

23.  Oligomenorrhea and amenorrhea are symptoms of hyperthyroidism

●      Yes

●      No

24.  A sudden increase of weight is a symptom of hypothyroidism

●      Yes

●      No

25.  Fatigability and sleepiness are manifestations of hypothyroidism

●      Yes

●      No

26.  Skin and hair dryness are symptoms of hypothyroidism

●      Yes

●      No

27.  Feeling cold in hot weather is a symptom of hypothyroidism

●      Yes

●      No

(IV) Respondents who had or suspect thyroid disease

28.  Do you have thyroid disease?

●      Yes

●      No

29.  If yes, what is the complaint?

●      Hypothyroidism

●      Hyperthyroidism

●      Thyroid cancer

30.  At which age this problem started?

●      1 - 12 years

●      12 - 20 years

●      20 - 35 years

●      >35 years

31.  Do you consult a doctor for treatment?

●      yes

●      no

32.  What are the treatments you take?

●      Dietary

●      Medications

33.  If Q30 answer was NO, do you suspect that you have thyroid disease?

●      Yes

●      No

34.  Why do you suspect?

●      A relative had the disease

●      Weight loss

●      Laziness

●      Increased weight

●      Symptoms appeared

## References

[1] Alyahya A, AlNaim A, AlBahr AW, Almansour F, Elshebiny A (2021). Knowledge of thyroid disease manifestations and risk factors among residents of the Eastern Province, Saudi Arabia. Cureus.

[2] Abdulrahman Ibrahim AM (2018). Survey of awareness of thyroid disorders among the Riyadh population, central region of Saudi Arabia. Egypt J Hosp Med.

[3] Issa LF, Alotbi MF, Algethami RF, Alharthi AA, Algarni FA, Alharthi KA. (2021). Assessment of knowledge and attitude regarding thyroid disorders among population in Taif city, Saudi Arabia. J Pharm Res Int.

[4] Alhazmi A, Alkhaldi B, Alhazmi R, Alzahrani A, Damanhouri N (2020). A cross-sectional study to evaluate knowledge, attitude, and practice among Saudi patients with hypothyroidism in Makkah. IJMDC.

[5] Toft AD, Beckett GJ (2003). Thyroid function tests and hypothyroidism. BMJ.

[6] Ladenson PW, Singer PA, Ain KB (2000). American Thyroid Association guidelines for detection of thyroid dysfunction. Arch Intern Med.

[7] Heuck CC, Kanagasabapathy AS, Kaller A, Riesen W (2000). Diagnosis and monitoring of disease of the thyroid.

[8] Kannan S (2010). Knowledge, awareness and practices (KAP) among patients with hypothyroidism attending endocrine clinics of community hospitals in Chennai. Thyroid Research and Practice.

[9] Rai S, Sirohi S, Khatri AK, Dixit S, Saroshe S (2016). Assessment of knowledge and awareness regarding thyroid disorders among women of a cosmopolitan city of central India. Natl J Community Med.

[10] Alhawiti AM, Albalawi AS, Alghamdi AA (2018). Assessment of public knowledge regarding the differences between hyperthyroidism and hypothyroidism. Egypt J Hosp Med.

[11] Rashad NM, Samir GM (2020). Prevalence, risks, and comorbidity of thyroid dysfunction: a cross-sectional epidemiological study. Egypt J Hosp Med.

[12] Alqahtani SA (2021). Prevalence and characteristics of thyroid abnormalities and its association with anemia in Asir region of Saudi Arabia: a cross-sectional study. Clin Pract.

[13] (2022). Harrison's Principles of Internal Medicine, 21e. Harrison’s Principles of Internal Medicine.

[14] Raval AD, Sambamoorthi U (2012). Incremental healthcare expenditures associated with thyroid disorders among individuals with diabetes. J Thyroid Res.

[15] Al-Geffari M, Ahmad NA, Al-Sharqawi AH, Youssef AM, Alnaqeb D, Al-Rubeaan K (2013). Risk factors for thyroid dysfunction among type 2 diabetic patients in a highly diabetes mellitus prevalent society. Int J Endocrinol.

